# Inflammation Meets Ventricular Dynamics: Combined Hematologic and Radiologic Predictors of Shunt-Dependent Hydrocephalus in Endovascularly Treated Patients with Aneurysmal Subarachnoid Hemorrhage

**DOI:** 10.3390/diagnostics16162506

**Published:** 2026-08-09

**Authors:** Ali Arslan, Zeki Boga, Semih Kivanc Olguner, Zişan Nur Sürmelioğlu, Mustafa Emre Sarac, Mehmet Ozer, Ali Harmanoğullarından, Ali Sürmelioğlu, Vedat Acik, Yurdal Gezercan

**Affiliations:** 1 Department of Neurosurgery, Adana City Training and Research Hospital, University of Health Sciences, Adana 01230, Turkey; zekiboga2013@gmail.com (Z.B.); kivanc3olguner@hotmail.com (S.K.O.); emre_sarac@hotmail.com (M.E.S.); aliharmanoglu@gmail.com (A.H.); ali.surmeli.30@gmail.com (A.S.); vedatacik74@gmail.com (V.A.); gezercan@hotmail.com (Y.G.); 2 Department of Radiology, Adana City Training and Research Hospital, University of Health Sciences, Adana 01230, Turkey; elazisan@gmail.com; 3 Department of Neurosurgery, Niğde Training and Research Hospital, Niğde 51100, Turkey; mehmetozerdr@gmail.com

**Keywords:** subarachnoid hemorrhage, intracranial aneurysm, hydrocephalus, cerebrospinal fluid shunts, intraventricular hemorrhage, inflammation, tomography, X-Ray-computed

## Abstract

**Background/Objectives**: Shunt-dependent hydrocephalus remains a major complication following aneurysmal subarachnoid hemorrhage (aSAH). Although several clinical–radiological prediction models have been proposed, the additional contribution of inflammatory biomarkers remains unclear. This study evaluated whether the platelet-to-neutrophil ratio provides additional predictive value beyond established clinical and radiological predictors of shunt-dependent hydrocephalus after aSAH. **Materials and Methods**: This retrospective single-center cohort study included 418 consecutive patients with aSAH treated using endovascular techniques between 2017 and 2025. Clinical severity parameters, ventricular burden-related radiological findings, and admission hematological variables were analyzed. Independent predictors of shunt-dependent hydrocephalus were identified using multivariable logistic regression analysis. Model performance was assessed using receiver operating characteristic analysis, calibration assessment, sensitivity analyses, and bootstrap internal validation. **Results**: Shunt-dependent hydrocephalus developed in 75 patients (18%). Lower Glasgow Coma Scale scores, intraventricular hemorrhage, increased Evans index, greater third ventricle width, lower platelet-to-neutrophil ratio, and external ventricular drain requirement were independently associated with shunt dependency. Incorporation of the platelet-to-neutrophil ratio increased the area under the curve from 0.84 (95% CI: 0.79–0.89) to 0.88 (95% CI: 0.84–0.92; *p* = 0.018). Internal validation demonstrated preserved model performance and calibration. Sensitivity analyses demonstrated similar findings after exclusion of external ventricular drain placement. **Conclusions**: Platelet-to-neutrophil ratio is independently associated with shunt-dependent hydrocephalus after aSAH and may be considered an additional component of integrated clinical–radiological risk assessment.

## 1. Introduction

Aneurysmal subarachnoid hemorrhage (aSAH) is a severe neurovascular disorder associated with considerable mortality and morbidity [1]. Although important advances have been achieved in diagnostic and therapeutic approaches, secondary complications after aSAH still play a major role in determining clinical outcome [2,3]. Among these complications, hydrocephalus remains one of the most frequent and clinically relevant conditions encountered following aSAH [4]. In some patients, shunt-dependent hydrocephalus requiring permanent cerebrospinal fluid diversion may develop, which can adversely affect long-term neurological recovery. Therefore, early identification of patients at risk for developing shunt dependency is of considerable importance for patient management and clinical follow-up [5].

Clinical and radiological factors associated with the development of shunt-dependent hydrocephalus have been evaluated in numerous previous studies. Intraventricular hemorrhage, ventricular enlargement, poor neurological status, and the requirement for external ventricular drainage have consistently been reported as among the most important predictors [6,7,8]. In addition, radiological parameters reflecting ventricular blood burden and early cerebrospinal fluid circulation disturbance have also been shown to be closely associated with the development of hydrocephalus [6,9,10]. Based on these findings, several clinical-radiological prediction models and scoring systems have been developed to estimate the risk of shunt-dependent hydrocephalus during the early phase after aSAH [11,12,13]. Nevertheless, most currently available models rely predominantly on clinical and radiological parameters, while the predictive contribution of the systemic inflammatory response remains only partially understood [4].

The inflammatory response that develops after subarachnoid hemorrhage is thought to play an important role in early brain injury and disturbances of cerebrospinal fluid circulation. In particular, inflammatory activation triggered by intraventricular blood products has been suggested to contribute to fibrotic alterations within the arachnoid granulations, thereby impairing cerebrospinal fluid absorption [4,14,15]. To better understand these processes, increasing attention has recently been directed toward the relationship between hematological inflammatory markers and complications following aSAH, including vasospasm, poor neurological outcomes, delayed cerebral ischemia, and hydrocephalus [15,16,17]. However, most available studies have focused primarily on neutrophil-to-lymphocyte ratio, platelet-to-lymphocyte ratio, or composite inflammatory indices, and whether inflammatory biomarkers provide additional predictive value when integrated into established clinical-radiological prediction models remains uncertain [11,16,17,18].

Data regarding the platelet-to-neutrophil ratio (PNR) remain limited. Previous studies in aSAH have mainly evaluated neutrophil-to-lymphocyte ratio and platelet-to-lymphocyte ratio, while the relationship between PNR and shunt-dependent hydrocephalus has received comparatively little attention [16,17]. Previous studies on shunt-dependent hydrocephalus after aSAH have included patients treated with different aneurysm occlusion strategies [6,7,8,11]. Because treatment modality may act as a potential confounding factor when evaluating predictors of shunt dependency, the present study was restricted to patients undergoing endovascular treatment in order to reduce treatment-related heterogeneity.

In the present study, clinical, radiological, and hematological factors associated with shunt-dependent hydrocephalus after aSAH were evaluated together. Particular emphasis was placed on determining whether PNR provides incremental predictive value beyond established clinical and radiological predictors. Accordingly, radiological parameters including intraventricular hemorrhage, Evans index, and third ventricle width were analyzed together with variables reflecting clinical disease severity to evaluate whether the addition of PNR improves multivariable clinical-radiological risk assessment for shunt-dependent hydrocephalus.

## 2. Materials and Methods

### 2.1. Study Design and Patient Selection

Patients diagnosed with aneurysmal subarachnoid hemorrhage (aSAH) and treated at our institution between 1 June 2017 and 1 October 2025 were retrospectively reviewed. The study was conducted at a tertiary referral center routinely managing a large number of neurovascular cases. Clinical findings, laboratory parameters, and imaging data were obtained from medical records collected during standard patient care. Patients were eligible if they had at least six months of follow-up after hemorrhage or if the primary endpoint occurred within the first six months. Accordingly, patients admitted at the end of the study period were followed until completion of the six-month follow-up period. Patients who died before completing the six-month follow-up were retained in the primary analysis using the available clinical data and excluded only from the prespecified mortality sensitivity analysis. At our institution, patients with aSAH are routinely evaluated in the outpatient clinic at approximately 1, 3, and 6 months after discharge, and cranial computed tomography (CT) is commonly obtained during these visits as part of standard clinical follow-up.

Initially, all patients diagnosed with aSAH during the study period were retrospectively screened through hospital records. Among these patients, those diagnosed by cranial computed tomography and whose ruptured aneurysms were confirmed by digital subtraction angiography (DSA) were identified. To ensure methodological homogeneity, only patients treated using endovascular techniques were included. Accordingly, patients treated primarily with coil embolization, either by simple coiling, balloon-assisted coiling, or stent-assisted coiling, were evaluated. Patients who underwent microsurgical clipping or combined surgical and endovascular treatment, as well as those with non-aneurysmal subarachnoid hemorrhage, a prior history of hydrocephalus or shunt placement, active infection or sepsis at admission, hematological disease or malignancy, immunosuppressive therapy, or incomplete clinical, laboratory, or imaging data, were excluded from the study.

**Figure 1:**
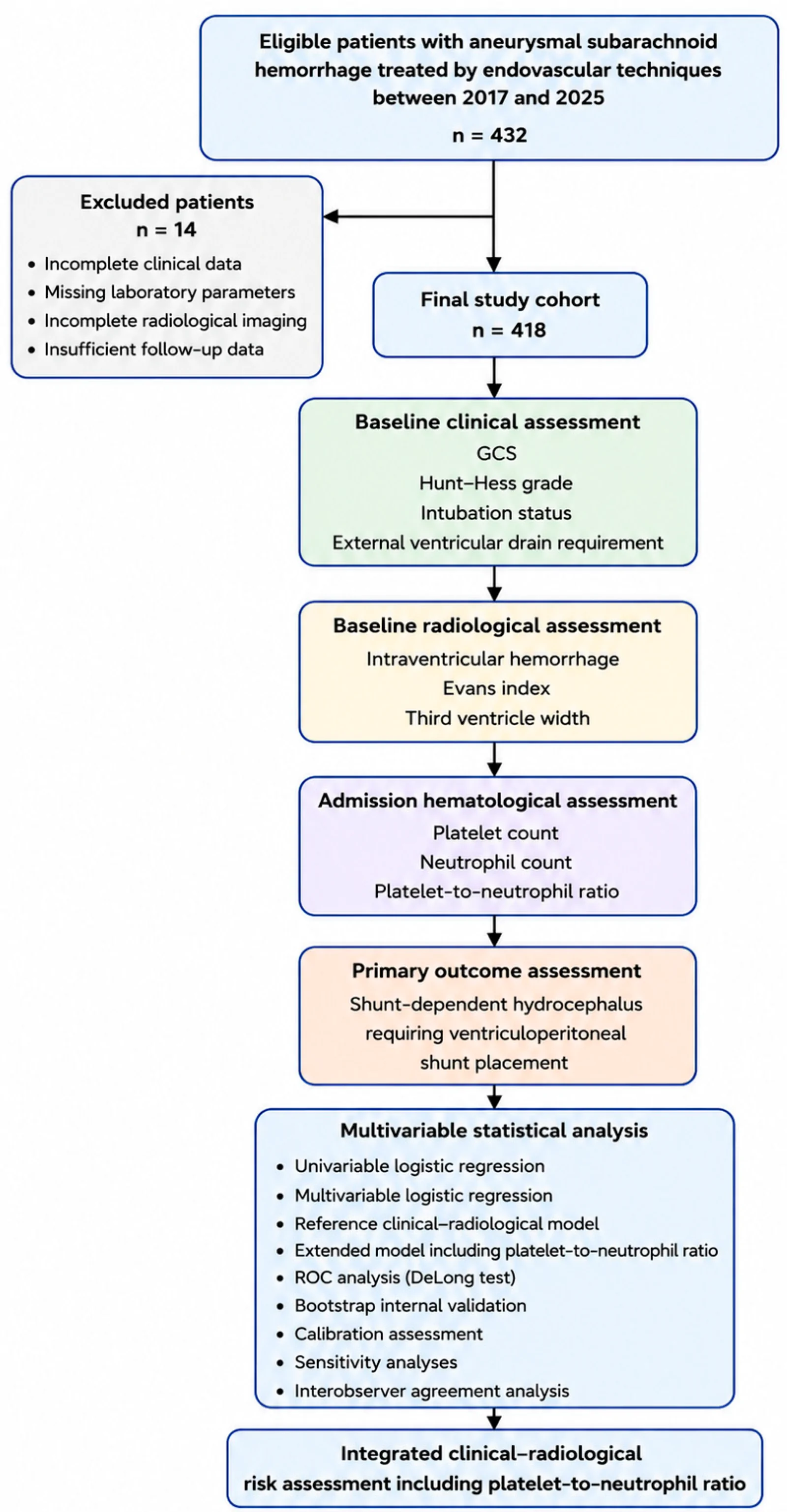
Overview of the study design and multivariable risk assessment workflow. aSAH: aneurysmal subarachnoid hemorrhage; GCS: Glasgow Coma Scale; IVH: intraventricular hemorrhage; PNR: platelet-to-neutrophil ratio.

During this process, a total of 432 patients were evaluated. Fourteen patients with incomplete clinical, laboratory, or imaging data were excluded, resulting in a final analytic cohort of 418 patients. The overall rate of missing data in the cohort was 3.2%, and all analyses were conducted using complete-case data. Baseline demographic and clinical characteristics were comparable between patients excluded because of missing data and those included in the final analysis. Details of patient selection and exclusion are presented in Figure 1. The methodological workflow of the study is presented in Figure 1, including patient selection, baseline clinical, radiological, and hematological evaluation, outcome assessment, and multivariable risk assessment analysis.

### 2.2. Outcome Definition

The primary endpoint of the study was defined as shunt-dependent hydrocephalus developing after aneurysmal subarachnoid hemorrhage (aSAH). Shunt-dependent hydrocephalus was considered hydrocephalus requiring ventriculoperitoneal shunt (VPS) placement for permanent cerebrospinal fluid diversion during the follow-up period. The decision for shunt placement was made by the responsible neurosurgical team based on combined clinical and radiological findings. No single radiological parameter was used in isolation when deciding on VPS placement. Progressive enlargement of the ventricular system on serial cranial computed tomography scans accompanied by clinical deterioration was considered indicative of shunt dependency. In patients with an external ventricular drain (EVD), the EVD was clamped during the weaning process, and patients were monitored with serial neurological examinations and follow-up cranial CT scans. Clinical deterioration or progressive ventricular enlargement during EVD weaning was considered an indication for permanent VPS placement. Temporary cerebrospinal fluid drainage with EVD alone was not accepted as an endpoint. Only patients who ultimately required permanent shunt placement were classified as having shunt-dependent hydrocephalus.

Except for patients who died before completing the six-month follow-up, all included patients had a minimum follow-up duration of six months. A six-month follow-up period was selected because shunt-dependent hydrocephalus after aSAH most commonly develops during the early post-hemorrhagic period, and this timeframe has been widely used in previous studies evaluating this outcome. Cases requiring VPS placement during this period were recorded as shunt-dependent hydrocephalus.

### 2.3. Radiological Evaluation and Parameters

Cranial computed tomography (CT) images obtained at the time of admission were retrospectively reviewed from the institutional imaging archive. Radiological evaluation focused on the presence of intraventricular hemorrhage (IVH), Evans index, and third ventricle width. These parameters were assessed to reflect intraventricular blood burden and ventricular enlargement. All imaging studies were reviewed in DICOM format using RadiAnt DICOM Viewer (version 2024.1; Medixant, Poznań, Poland). Radiological evaluations were performed on standard axial sections by two investigators, with the imaging studies divided between the two investigators for primary radiological assessment. All measurements were obtained using the electronic measurement tools provided by the software.

**Figure 2:**
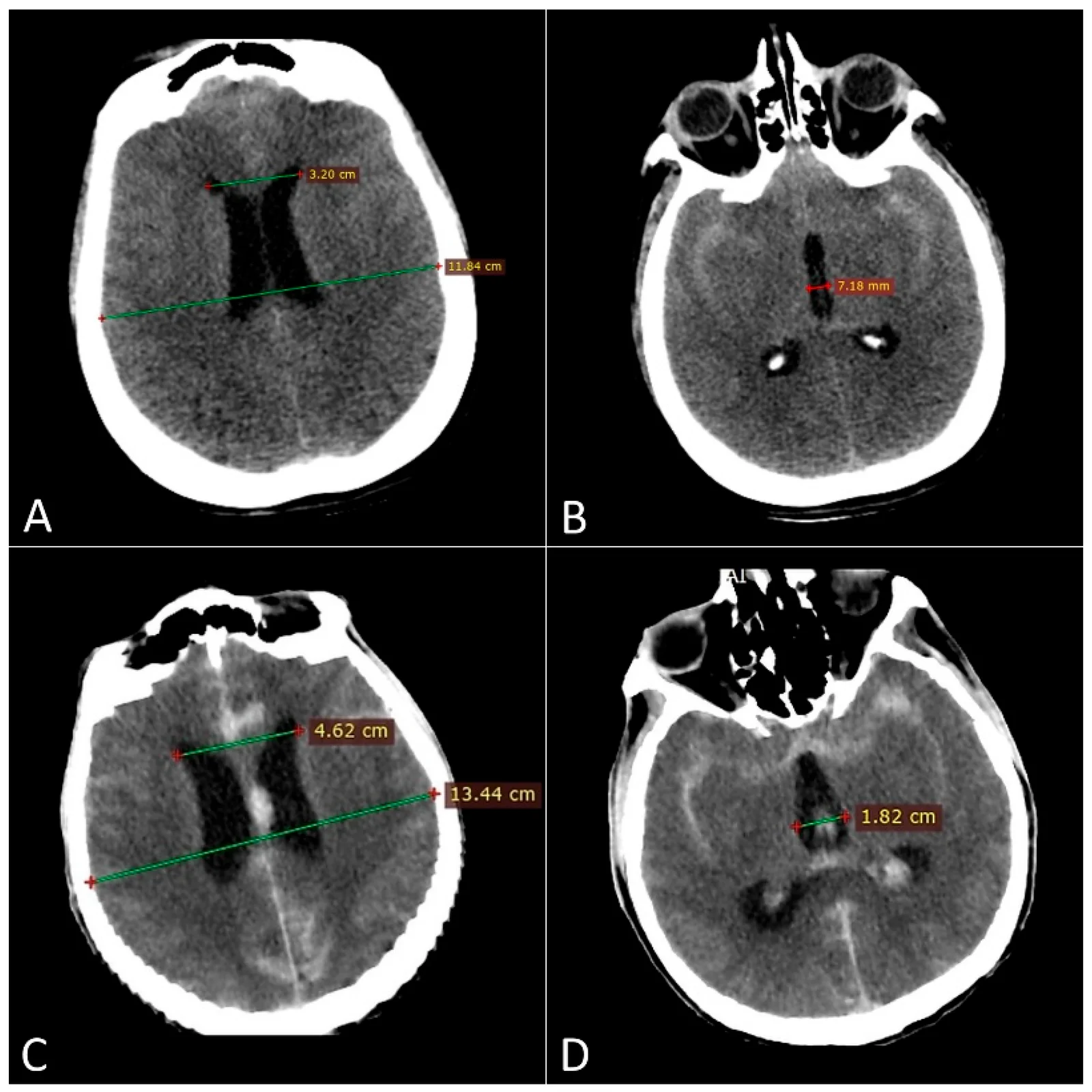
Representative axial cranial CT images demonstrating ventricular measurements in patients with low- and high-risk profiles for shunt-dependent hydrocephalus following aneurysmal subarachnoid hemorrhage. (**A**) Evans index measurement in a low-risk patient. (**B**) Third ventricular width measurement in a low-risk patient. (**C**) Evans index measurement in a high-risk patient with more prominent ventricular dilatation. (**D**) Third ventricular width measurement in a high-risk patient.

The presence of intraventricular hemorrhage (IVH) was recorded as present or absent according to standard definitions. To quantitatively assess ventricular enlargement, Evans index and third ventricle width were measured. These measurements were recorded as continuous variables. Evans index was defined as the ratio of the maximum width of the frontal horns to the maximum intracranial diameter measured on the same axial section. Third ventricle width was measured in millimeters on the axial section demonstrating the widest dimension of the third ventricle. Representative axial CT images demonstrating ventricular measurements are shown in Figure 2.

All radiological measurements were obtained from the admission CT scan. Shunt decisions were based on the overall clinical and radiological assessment and not on a single radiological measurement.

To evaluate interobserver agreement, a third neurosurgeon, blinded to the clinical outcomes, independently re-evaluated imaging studies from a randomly selected subset of 60 patients from the entire cohort. Cohen’s kappa coefficient was used for categorical variables, whereas the intraclass correlation coefficient (ICC) based on a two-way random-effects model with absolute agreement was used for continuous measurements. For ICC analysis, the single-measurement absolute-agreement model was applied. In cases of disagreement between measurements, final values were determined by joint consensus evaluation.

### 2.4. Hematological and Clinical Variables

Hematological data were obtained from complete blood count analyses performed at admission before any surgical or endovascular intervention. Platelet count and absolute neutrophil count were recorded, and PNR was calculated by dividing these values. All hematological measurements were performed using the same laboratory analysis system. Clinical data were retrospectively obtained from the hospital information management system and patient medical records. Recorded variables included age, sex, and neurological status at admission. All patients were monitored in the neurosurgical intensive care unit following admission. Neurological status at presentation was assessed using the Glasgow Coma Scale, and disease severity was recorded according to the Hunt–Hess classification. Intubation status at admission was recorded as either present or absent and was accepted as an indicator of clinical severity. Accompanying systemic diseases were assessed using patient records. Endovascular treatment characteristics, including simple coiling, balloon-assisted coiling, and stent-assisted coiling, were reviewed and recorded. In addition, treatment-related factors such as timing of treatment and procedure-related complications were reviewed and recorded. External ventricular drain placement, timing of insertion, and duration of drainage were additionally documented.

### 2.5. Data Collection

Clinical, hematological, and radiological data used in the study were retrospectively obtained from hospital information system records and imaging archives generated during routine patient care. All clinical, laboratory, and imaging data were derived from routinely maintained institutional records and imaging archives available through the hospital information system. Clinical, laboratory, and imaging data belonging to the same patient were integrated and used for analysis. Data entries were reviewed for accuracy. Before statistical analysis, all variables were organized according to predefined criteria and appropriately recorded as categorical or continuous variables.

### 2.6. Statistical Analysis

Statistical analyses were performed using IBM SPSS Statistics (version 26.0; IBM Corp., Armonk, NY, USA) and R software (version 4.3.2; R Foundation for Statistical Computing, Vienna, Austria). Because of the retrospective study design, no formal sample size calculation was performed. Instead, all consecutive eligible patients meeting the predefined inclusion criteria during the study period were included in the analysis. Distribution of continuous variables was assessed using histograms and Q–Q plots. Normally distributed variables were presented as mean ± standard deviation, whereas non-normally distributed variables were expressed as median and interquartile range. Categorical variables were presented as counts and percentages.

Comparisons were performed between patients who developed shunt-dependent hydrocephalus and those who did not. Categorical variables were analyzed using the chi-square test or Fisher’s exact test, while continuous variables were analyzed using Student’s *t*-test or Mann–Whitney U test according to data distribution.

Logistic regression analysis was used to identify factors associated with the development of shunt-dependent hydrocephalus. Initially, univariable logistic regression analyses were performed. Variables demonstrating a *p*-value < 0.10 in univariable analyses, together with variables considered clinically relevant based on prior literature and biological plausibility, were entered into the multivariable logistic regression model using a forced-entry approach. A multivariable logistic regression model integrating clinical, radiological, and hematological variables was constructed to evaluate the incremental contribution of PNR to clinical–radiological risk assessment for shunt-dependent hydrocephalus. Model performance was evaluated by comparing a reference model including clinical and radiological variables with an extended model incorporating PNR. Areas under the receiver operating characteristic (ROC) curves were used for model comparison, and differences between model performances were statistically assessed using the DeLong test.

Continuous variables were used in the analysis as continuous measures without categorization. For clinical interpretability and directional consistency, GCS was reverse-coded using the transformation (15 − GCS), such that the regression coefficient and odds ratio represented each 1-point decrease in GCS. Similarly, PNR was reverse-coded and scaled using the transformation (−PNR/5), such that the regression coefficient and odds ratio represented each 5-unit decrease in PNR. Multicollinearity between variables was evaluated with variance inflation factor (VIF) analysis, and no clinically meaningful multicollinearity was observed in the multivariable model. External ventricular drain (EVD) placement was additionally included as a possible confounding variable. Because EVD placement may reflect both disease severity and a management-related intermediate variable, an additional sensitivity analysis excluding EVD from the multivariable model was performed to evaluate the robustness of the findings. In addition, to evaluate the potential influence of mortality as a competing event, a sensitivity analysis was performed after excluding patients who died before adequate follow-up for assessment of shunt dependency. The multivariable logistic regression model was then refitted, and the resulting effect estimates were compared with those of the primary analysis. Logistic regression results were reported as odds ratios (ORs) with 95% confidence intervals (CIs). Model discrimination was assessed using the area under the ROC curve, and AUC values were presented together with 95% confidence intervals.

Internal validation of the multivariable logistic regression model was performed using bootstrap resampling with 1000 iterations to estimate optimism-corrected model performance. Model calibration was evaluated using bootstrap-corrected calibration plots and the Hosmer–Lemeshow goodness-of-fit test. Calibration performance was assessed by comparing observed and predicted probabilities across predefined probability levels.

No time-to-event analysis regarding the timing of shunt requirement was performed, and outcomes were analyzed as a binary endpoint. To reduce the risk of overfitting, the number of variables included in the multivariable model was restricted according to the number of outcome events. The events-per-variable ratio was additionally considered during multivariable model development to improve model stability and reduce the likelihood of overfitting. In all analyses, a two-sided *p*-value < 0.05 was considered statistically significant.

### 2.7. Ethical Approval and Informed Consent Statement

The study protocol was reviewed and approved by the Clinical Research Ethics Committee of Adana City Training and Research Hospital (Meeting No: 25; Decision No: 1300; Approval Date: 30 April 2026). The retrospective review and analysis of routinely collected clinical, laboratory, and imaging data were performed after ethics committee approval. All procedures were conducted in accordance with the ethical principles of the Declaration of Helsinki. Due to the retrospective design of the study and the use of anonymized routinely collected clinical data, the requirement for additional study-specific informed consent was waived by the ethics committee. Written informed consent regarding hospitalization, treatment procedures, and the use of anonymized clinical data for scientific and research purposes had been obtained from all patients or their legal representatives in accordance with institutional regulations.

## 3. Results

### 3.1. Study Population and Baseline Characteristics

A total of 418 patients constituted the final analytic cohort. The median age of the cohort was 61 years (52–70), with a slight predominance of female patients (57%). The median Glasgow Coma Scale score was 13 (10–15), and most patients were classified within the lower Hunt–Hess grades (I–II, 55%). In addition, 25% of patients required intubation at admission. Intraventricular hemorrhage was present in 38% of the cohort. During follow-up, shunt-dependent hydrocephalus developed in 18% of patients. Endovascular treatment was performed using simple coiling in 334 patients (79.9%), balloon-assisted coiling in 63 patients (15.1%), and stent-assisted coiling in 21 patients (5.0%). Detailed demographic, clinical, hematological, and radiological characteristics of the study population are presented in Table 1.

### 3.2. Comparison Between Patients with and Without Shunt-Dependent Hydrocephalus

Comparative analyses between patients who developed shunt-dependent hydrocephalus and those who did not are presented in Table 2. Patients who developed shunt-dependent hydrocephalus had lower GCS scores and more frequently demonstrated higher Hunt–Hess grades and a requirement for intubation at admission. The prevalence of intraventricular hemorrhage, Evans index values, and third ventricle width measurements were all significantly higher in the shunt-dependent group. Platelet-to-neutrophil ratio also differed significantly between the two groups. Among the 75 patients who developed shunt-dependent hydrocephalus, 49 (65%) had undergone external ventricular drain placement during the acute phase, whereas 26 (35%) developed delayed shunt-dependent hydrocephalus during follow-up without requiring prior EVD placement.

### 3.3. Multivariable Logistic Regression Analysis

Multivariable logistic regression analysis was performed to identify independent factors associated with the development of shunt-dependent hydrocephalus. In the multivariable model, lower GCS scores, the presence of intraventricular hemorrhage, increased Evans index, greater third ventricle width, lower PNR, and external ventricular drain placement were independently associated with shunt-dependent hydrocephalus (Table 3). Platelet-to-neutrophil ratio remained independently associated with shunt dependency even after adjustment for clinical variables, radiological parameters, and EVD placement. To facilitate model reproducibility, the regression coefficients and model intercept are presented in Table 3.

### 3.4. Model Performance and Internal Validation

**Figure 3:**
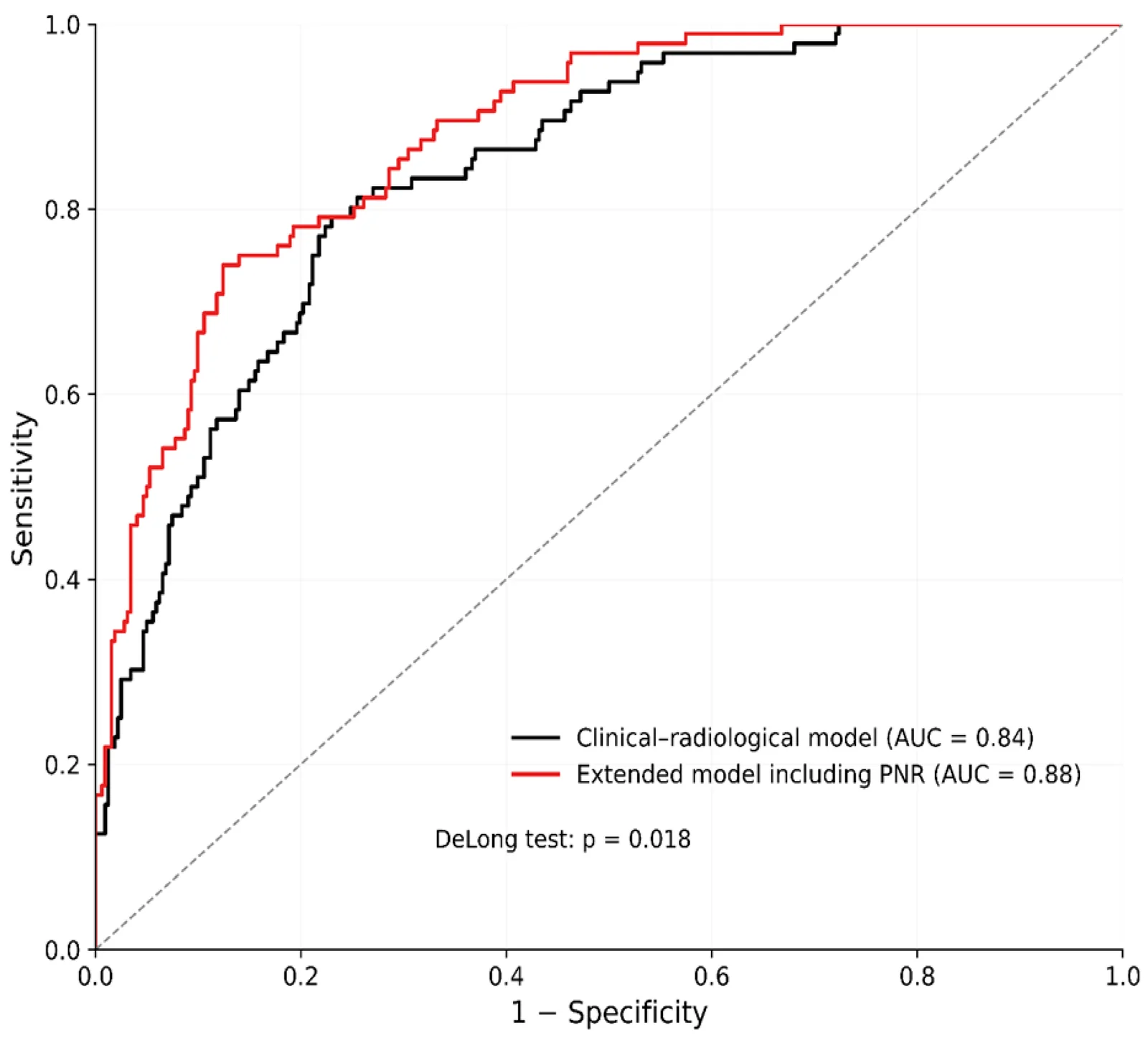
Receiver operating characteristic (ROC) curves comparing the clinical–radiological reference model and the extended model including platelet-to-neutrophil ratio. The dashed diagonal line represents the chance line.

The discriminative performance of the reference model including clinical and radiological variables yielded an area under the receiver operating characteristic curve (AUC) of 0.84 (95% CI: 0.79–0.89). After incorporation of PNR as a hematological parameter, the AUC increased to 0.88 (95% CI: 0.84–0.92). The difference between the two models was statistically significant according to the DeLong test (*p* = 0.018). ROC curves for the reference and extended models are presented in Figure 3.

**Figure 4:**
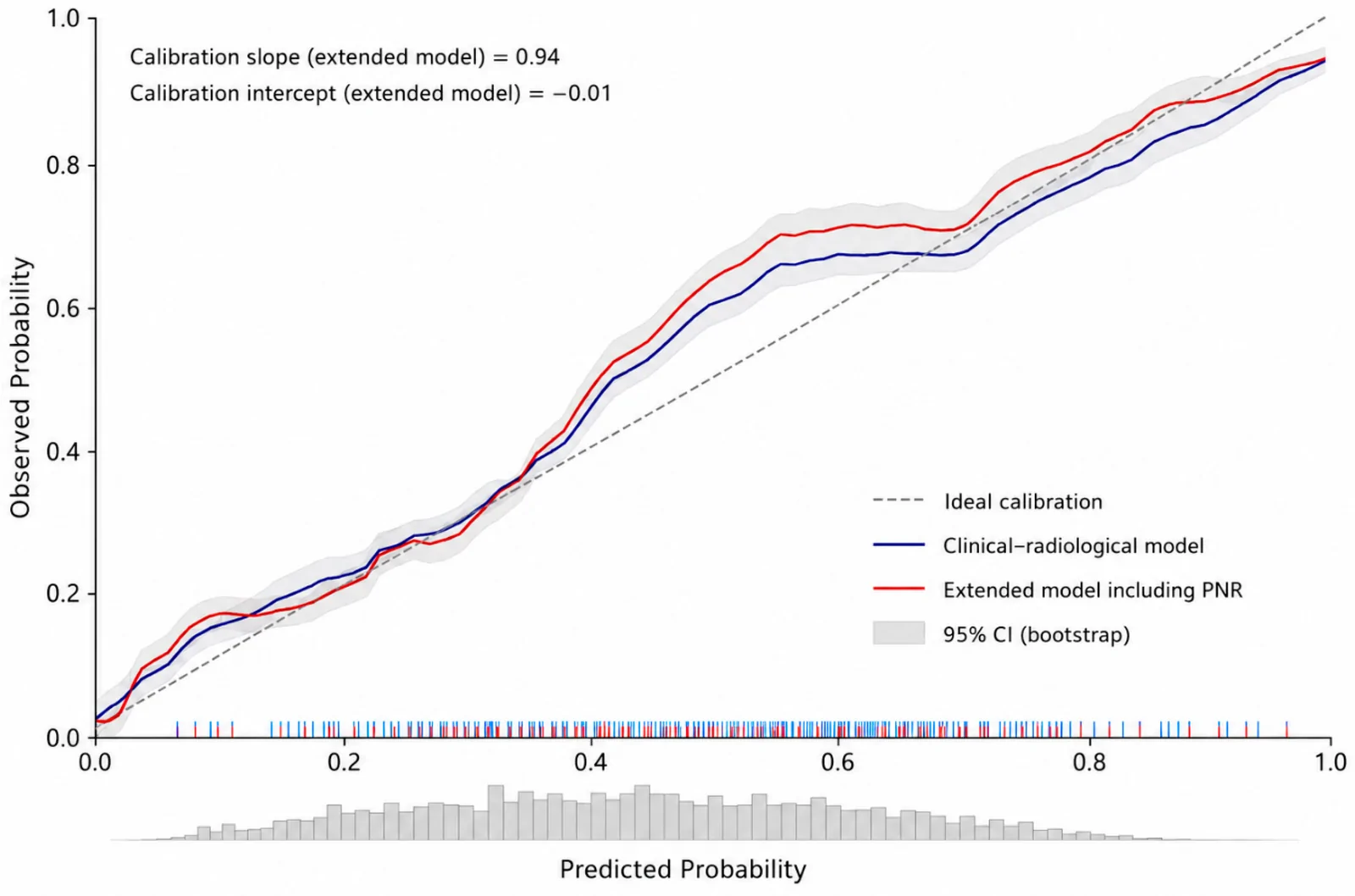
Bootstrap-corrected calibration curves for the clinical–radiological reference model and the extended model including platelet-to-neutrophil ratio.

Bootstrap resampling for internal validation showed that model performance was largely maintained after optimism correction, with only a small decline observed. The optimism-corrected AUC of the extended model was 0.86. Calibration analysis demonstrated good concordance between predicted and observed probabilities. The calibration slope and intercept for the extended model were 0.94 and −0.01, respectively, suggesting no substantial overfitting or systematic risk overestimation. The Hosmer–Lemeshow goodness-of-fit test was not statistically significant (*p* = 0.41). To further verify the internal consistency of the reported logistic regression equation, the final equation was applied to all 418 patients included in the primary cohort, and the mean predicted probability was calculated. The resulting mean predicted probability was 19.1%, which closely corresponded to the observed event rate of 17.9% (75/418), confirming the internal consistency of the reported regression equation. Calibration plots for the reference and extended models are shown in Figure 4.

Additional sensitivity analyses excluding external ventricular drain placement from the multivariable model demonstrated similar findings. Platelet-to-neutrophil ratio remained independently associated with shunt-dependent hydrocephalus (OR: 1.20, 95% CI: 1.04–1.39; *p* = 0.018), while overall model discrimination remained largely preserved (AUC: 0.85).

### 3.5. Sensitivity Analysis

To evaluate the potential influence of mortality as a competing event, a sensitivity analysis was performed after excluding patients who died before adequate follow-up for assessment of shunt dependency (Table 4). A total of 18 patients were excluded, resulting in a final sensitivity cohort of 400 patients, including 75 patients who developed shunt-dependent hydrocephalus. Platelet-to-neutrophil ratio remained significantly associated with shunt-dependent hydrocephalus (OR: 1.20, 95% CI: 1.04–1.39; *p* = 0.018). The estimated odds ratios for the remaining variables were comparable with those observed in the primary analysis.

### 3.6. Interobserver Agreement

Interobserver agreement for radiological evaluations was assessed. The Cohen’s kappa coefficient for the presence of intraventricular hemorrhage was calculated as 0.85. For continuous radiological measurements, intraclass correlation coefficient values were 0.89 for Evans index and 0.87 for third ventricle width.

## 4. Discussion

In the present study, clinical, radiological, and hematological factors related to the development of shunt-dependent hydrocephalus after aneurysmal subarachnoid hemorrhage were assessed together within a combined predictive model. Multivariable analysis demonstrated that lower GCS scores, the presence of intraventricular hemorrhage, increased Evans index, greater third ventricle width, and external ventricular drain placement were associated with shunt-dependent hydrocephalus. In addition, PNR maintained its independent association with shunt dependency even after adjustment for established clinical and radiological predictors. Incorporation of PNR into the reference clinical–radiological model resulted in a modest but statistically significant improvement in discriminative performance, while calibration performance and internal validation findings remained preserved.

These findings suggest that shunt-dependent hydrocephalus after aSAH may be influenced not only by ventricular burden and clinical disease severity, but also by systemic inflammatory activity. Importantly, the principal contribution of the present study lies in providing early evidence that PNR may be independently associated with shunt-dependent hydrocephalus after adjustment for established clinical and radiological predictors.

Previous studies examining the clinical and radiological predictors of shunt-dependent hydrocephalus have consistently reported poor neurological status, intraventricular hemorrhage burden, and ventricular enlargement as major determinants of permanent cerebrospinal fluid diversion after aSAH [7,9]. Consistent with these findings, patients who developed shunt-dependent hydrocephalus in our cohort had lower GCS scores, higher Hunt–Hess grades, more frequent intraventricular hemorrhage, and greater ventricular enlargement at baseline. In multivariable analysis, intraventricular hemorrhage, Evans index, and third ventricle width remained independently associated with shunt dependency, supporting the view that early ventricular burden and impaired cerebrospinal fluid circulation have central roles in this process.

These findings support the concept that early ventricular burden may play a central role in the development of shunt-dependent hydrocephalus after aSAH. Intraventricular hemorrhage may contribute to the development of hydrocephalus not only by mechanically obstructing cerebrospinal fluid pathways, but also through secondary inflammatory mechanisms that affect cerebrospinal fluid absorption and ventricular dynamics [4,19]. Similarly, more pronounced ventricular enlargement at baseline may indicate early impairment of cerebrospinal fluid circulation and reduced compensatory adaptation of the ventricular system [19]. Consistent with these observations, previous clinical–radiological prediction models have similarly identified intraventricular hemorrhage burden and ventricular enlargement as major components associated with shunt dependency [10,19].

The observed independent association for external ventricular drain placement should be approached cautiously. The requirement for EVD probably reflects higher initial disease severity, a larger ventricular blood load, and more substantial impairment of cerebrospinal fluid circulation rather than a direct causal effect of the procedure itself. Supporting this interpretation, sensitivity analyses excluding EVD from the multivariable model showed that the main findings remained unchanged, further supporting the stability of the association between PNR and shunt dependency.

One of the important findings of the present study was that our results provide early evidence that PNR may be independently associated with shunt-dependent hydrocephalus after adjustment for established predictors. Previous studies in patients with aSAH have primarily focused on inflammatory biomarkers such as the neutrophil-to-lymphocyte ratio and platelet-to-lymphocyte ratio, whereas PNR has received comparatively little attention [16,17]. Accumulating evidence suggests that the inflammatory response following subarachnoid hemorrhage contributes to early brain injury and subsequent neuroinflammatory cascades [15]. In particular, inflammatory activation related to intraventricular blood products has been proposed to impair cerebrospinal fluid absorption through fibrosis and dysfunction of the arachnoid granulations [4,19]. In this setting, PNR may serve as a readily available biomarker reflecting the systemic thromboinflammatory response associated with hydrocephalus development after aSAH [15,17]. Although PNR remained independently associated with shunt-dependent hydrocephalus, it should not be interpreted as a stand-alone predictor. Rather, it may provide additional information when interpreted together with established clinical and radiological predictors. Nevertheless, these findings should not be interpreted as evidence of a direct causal relationship, and PNR may instead represent a surrogate marker reflecting overall disease severity and neuroinflammatory burden after aSAH.

Most previous studies investigating inflammatory biomarkers in aSAH have evaluated hematological parameters in isolation [16,17]. In contrast, the present study assessed inflammatory biomarkers together with established clinical and radiological variables within an integrated predictive framework. Incorporation of PNR into the reference clinical–radiological model was associated with a modest but statistically significant improvement in discriminative performance while calibration remained preserved after internal validation. Importantly, the magnitude of this improvement should not be interpreted as representing a major shift in predictive capability, but rather as incremental refinement of risk stratification performance. Accordingly, inflammatory biomarkers may provide additional information when interpreted alongside established clinical and radiological predictors rather than as isolated indicators of shunt dependency risk.

An important strength of the present study is that model evaluation incorporated assessment of both discrimination and calibration. The extended model demonstrated good agreement between observed and predicted probabilities, with calibration slope and intercept values suggesting preserved calibration performance and no evidence of substantial systematic prediction bias. Furthermore, the limited decline in performance observed after bootstrap internal validation reduces the likelihood that the reported findings were primarily driven by model overfitting.

Although several clinical–radiological prediction models for shunt dependency after aSAH have previously been proposed, inflammatory biomarkers have generally been incorporated only to a limited extent within these frameworks [12,13,18]. In the present study, hematological inflammatory parameters were evaluated together with established clinical and radiological predictors within an integrated predictive framework, and model performance was assessed in terms of both discrimination and calibration. This integrated approach may contribute to improved risk stratification while maintaining the established clinical–radiological assessment strategy.

Shunt-dependent hydrocephalus remains one of the major complications influencing both the clinical course and long-term neurological outcomes after aneurysmal subarachnoid hemorrhage [5,7]. Therefore, early recognition of patients at increased risk for permanent cerebrospinal fluid diversion is clinically important for optimizing surveillance and management strategies [8,18]. In this context, the findings of the present study provide early evidence that incorporating PNR into conventional clinical–radiological assessment may contribute to risk stratification in patients at higher risk of shunt dependency during the early phase after aSAH. Such information may support closer clinical surveillance and follow-up rather than alter treatment decisions.

Several limitations of the present study should be acknowledged. Because of the retrospective single-center design, the findings may have been influenced by selection bias and center-specific management practices. Although internal validation was performed using bootstrap resampling, the absence of an independent external validation cohort limits the generalizability of the study findings and the proposed multivariable risk assessment approach. Hematological parameters were evaluated only at admission, preventing assessment of temporal changes in systemic inflammatory activity over time after hemorrhage. Likewise, no time-to-event analysis regarding the timing of shunt dependency was performed, limiting evaluation of the temporal dynamics underlying hydrocephalus development after aSAH. In addition, although external ventricular drain placement was incorporated into the multivariable model because of its close association with disease severity and cerebrospinal fluid circulation disturbance, EVD requirement may also partially reflect treatment-related clinical decision-making.

Despite these limitations, the study also has several important strengths. The relatively large cohort and the use of a standardized radiological assessment protocol helped maintain consistency across imaging analyses, further supported by evaluation of interobserver agreement. In addition to discrimination performance, the multivariable model was assessed using calibration analysis and internal validation to better evaluate its stability. Another notable aspect of the study was the assessment of inflammatory biomarkers together with established clinical and radiological predictors within the same predictive framework. Future multicenter validation studies and longitudinal evaluation of inflammatory markers at multiple time points may further clarify the clinical applicability and biological implications of these findings.

## 5. Conclusions

Platelet-to-neutrophil ratio is independently associated with shunt-dependent hydrocephalus after aneurysmal subarachnoid hemorrhage, even after adjustment for established clinical and radiological predictors. Incorporation of PNR into a conventional clinical–radiological model modestly improves predictive performance while preserving calibration after internal validation. These findings provide early evidence that PNR may be considered an additional component of an integrated risk assessment strategy for shunt dependency after aSAH. Further prospective studies are needed to confirm these findings.

## Figures and Tables

**Table 1:** Baseline demographic, clinical, hematological, and radiological characteristics of the study population.

Variable	Overall (*n* = 418)
Age, years *	61 (IQR: 52–70)
Female sex, *n* (%)	238 (57%)
GCS *	13 (IQR: 10–15)
Hunt-Hess grade, *n* (%)	
I–II	230 (55%)
III–V	188 (45%)
Intubation, *n* (%)	105 (25%)
External ventricular drain, *n* (%)	107 (26%)
Intraventricular hemorrhage, *n* (%)	159 (38%)
Evans index	0.32 ± 0.05
Third ventricle width (mm)	6.0 ± 1.5
Platelet count (×10^9^/L)	248 ± 65
Neutrophil count (×10^9^/L)	8.0 ± 3.0
Platelet-to-neutrophil ratio	31 ± 12
Shunt-dependent hydrocephalus, *n* (%)	75 (18%)

* Continuous variables are presented as median (IQR) or mean ± standard deviation, as appropriate. Categorical variables are presented as *n* (%). GCS: Glasgow Coma Scale, IQR: interquartile range.

**Table 2:** Comparison of baseline characteristics between patients with and without shunt-dependent hydrocephalus.

Variable	No Shunt (*n* = 343)	Shunt (*n* = 75)	*p*-Value
Age, years *	60 (51–69)	64 (56–72)	0.041
Female sex, *n* (%)	192 (56)	46 (61)	0.438
GCS *	14 (12–15)	11 (8–13)	<0.001
Hunt–Hess grade, *n* (%)			<0.001
I–II	205 (60)	25 (33)	
III–V	138 (40)	50 (67)	
Intubation, *n* (%)	67 (20)	38 (51)	<0.001
Intraventricular hemorrhage, *n* (%)	108 (31)	51 (68)	<0.001
Evans index	0.30 ± 0.04	0.37 ± 0.05	<0.001
Third ventricle width (mm)	5.4 ± 1.2	7.3 ± 1.6	<0.001
Platelet count (×10^9^/L)	251 ± 64	236 ± 69	0.081
Neutrophil count (×10^9^/L)	7.7 ± 2.8	9.1 ± 3.4	0.012
Platelet-to-neutrophil ratio	33 ± 11	24 ± 10	<0.001
External ventricular drain, *n* (%)	58 (17)	49 (65)	<0.001

* Continuous variables are presented as median (IQR) or mean ± standard deviation, as appropriate. Categorical variables are presented as *n* (%). GCS: Glasgow Coma Scale.

**Table 3:** Multivariable logistic regression analysis for shunt-dependent hydrocephalus.

Variable	β Coefficient	Adjusted OR	95% CI	*p*-Value
GCS, per 1-point decrease	0.168	1.18	1.06–1.32	0.003
Intraventricular hemorrhage	0.838	2.31	1.28–4.17	0.005
Evans index, per 0.01 increase	0.145	1.16	1.08–1.25	<0.001
Third ventricle width, per 1 mm increase	0.276	1.32	1.12–1.56	0.001
Platelet-to-neutrophil ratio, per 5-unit decrease	0.237	1.27	1.10–1.47	0.001
External ventricular drain	1.006	2.74	1.47–5.12	0.002
Intercept (Constant)	−5.200	—	—	—

Regression coefficients (β), adjusted odds ratios, and the model intercept are presented to facilitate reproducibility of the final multivariable logistic regression model. Continuous variables were entered into the model as continuous measures without categorization. GCS and platelet-to-neutrophil ratio were reverse-coded to represent each 1-point and 5-unit decrease, respectively. IVH and EVD were coded as 0 = absent and 1 = present. The final multivariable logistic regression model was defined as: Logit(P) = −5.200 + 0.168 × (15 − GCS) + 0.838 × (IVH) + 0.145 × (Evans index/0.01) + 0.276 × (Third ventricle width [mm]) + 0.237 × (−PNR/5) + 1.006 × (EVD) where P denotes the predicted probability of shunt-dependent hydrocephalus. The corresponding predicted probability was calculated as: P = exp[Logit(P)]/{1 + exp[Logit(P)]}.

**Table 4:** Sensitivity analysis after exclusion of patients who died before adequate follow-up for assessment of shunt dependency.

Variable	Primary Model Adjusted OR (95% CI)	*p*-Value	Mortality Sensitivity Analysis Adjusted OR (95% CI)	*p*-Value
GCS, per 1-point decrease	1.18 (1.06–1.32)	0.003	1.16 (1.05–1.29)	0.005
Intraventricular hemorrhage	2.31 (1.28–4.17)	0.005	2.24 (1.23–4.06)	0.006
Evans index, per 0.01 increase	1.16 (1.08–1.25)	<0.001	1.14 (1.07–1.23)	<0.001
Third ventricle width, per 1 mm increase	1.32 (1.12–1.56)	0.001	1.30 (1.11–1.53)	0.002
Platelet-to-neutrophil ratio, per 5-unit decrease	1.27 (1.10–1.47)	0.001	1.20 (1.04–1.39)	0.018
External ventricular drainage (EVD)	2.74 (1.47–5.12)	0.002	1.98 (1.05–3.74)	0.034

Odds ratios are adjusted for the variables included in each model. Continuous variables were entered into the model without categorization. The mortality sensitivity analysis excluded patients who died before adequate follow-up for assessment of shunt dependency. CI: confidence interval; GCS: Glasgow Coma Scale; OR: odds ratio.

## Data Availability

The datasets generated and/or analyzed during the current study are available from the corresponding author upon reasonable request. Public sharing of the data is restricted because of patient confidentiality and institutional policies. Access to anonymized data may be provided to qualified researchers following approval by the Clinical Research Ethics Committee of Adana City Training and Research Hospital.

## Abbreviations

aSAH	Aneurysmal Subarachnoid Hemorrhage
AUC	Area Under the Curve
CI	Confidence Interval
CT	Computed Tomography
DSA	Digital Subtraction Angiography
EVD	External Ventricular Drain
GCS	Glasgow Coma Scale
ICC	Intraclass Correlation Coefficient
IQR	Interquartile Range
IVH	Intraventricular Hemorrhage
OR	Odds Ratio
PNR	Platelet-to-Neutrophil Ratio
ROC	Receiver Operating Characteristic
SD	Standard Deviation
VIF	Variance Inflation Factor
VPS	Ventriculoperitoneal Shunt
